# Case Report: A Child with Gross Hematuria and the Importance of Travel History

**DOI:** 10.3389/fped.2018.00014

**Published:** 2018-02-05

**Authors:** Ibrahim F. Shatat

**Affiliations:** ^1^Pediatric Nephrology and Hypertension, Sidra Medicine, Doha, Qatar; ^2^Weill Cornell Medical College in Qatar, Doha, Qatar; ^3^Medical University of South Carolina, Charleston, SC, United States

**Keywords:** gross hematuria, *Schistosoma haematobium*, pediatrics, travel health, schistosomiasis

## Abstract

We report a case of a 5-year-old girl who presented with a 3-month-long history of gross hematuria. She underwent an extensive laboratory workup (including an automated urine microscopy) and a kidney biopsy, all of which were within normal limits. While being prepared for a cystoscopy and more advanced imaging of the urinary tract, the family mentioned history of travel to a schistosomiasis endemic area prompting a more thorough ova and parasite examination of the urine. Urine microscopy confirmed the diagnosis of *Schistosoma hematobium*.

## Background

**Figure 1:**
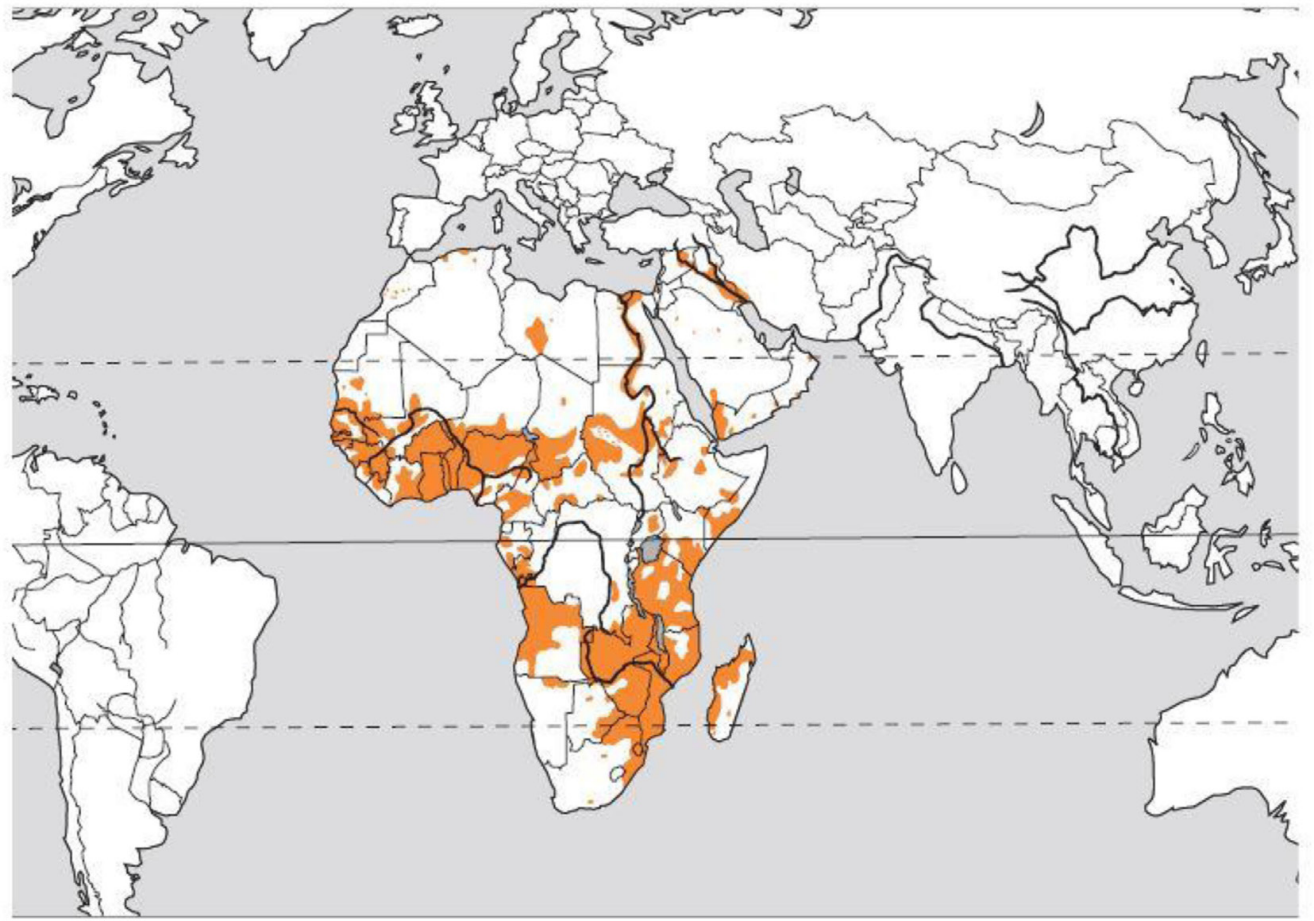
Distribution of the blood fluke *Schistosoma hematobium*. Courtesy of Dr. Bruno Gryseels, Institute of Tropical Medicine (1), modified from the original publication (2).

Gross hematuria—the presence of blood in the urine on visual inspection—is a relatively common presenting symptom in the pediatric nephrology clinic. The differential diagnosis of red urine includes, but is not limited to, infectious, glomerular etiology (glomerulonephritis), kidney stones, history of trauma, and in rare cases factitious or pseudohematuria. Schistosomiasis, also known as snail fever and bilharzia, is a disease caused by parasitic flatworms called schistosomes. It is rarely seen in the Western countries but is prevalent in tropical and subtropical areas, Figure 1 (1). With population movements, migration, and ease of travel across contents, schistosomiasis has been introduced to new areas. There are two major forms of schistosomiasis (intestinal and urological) caused by six main species; *Schistosoma hematobium* is usually the species associated with urogenital infection.

## Case Presentation

A 5-year-old girl presented to our pediatric nephrology and hypertension clinic at Sidra Medicine (Qatar) with a 3-month history of tea-colored urine. She was treated with antibiotics by a general practitioner for a presumptive urinary tract infection. The severity of her gross hematuria was waxing and waning, persistent, and throughout the urination. While on occasions, the urine was reddish, it was tea-colored on other occasions. There were no associated symptoms of dysuria, urgency, frequency, or stone passage. The family and the child denied any history of preceding trauma, easy bruising, foul smelling urine, and recurrent fever or urinary tract infections. There was no preceding history of throat or skin infection and no history of generalized swelling or change in the urine output. Her examination was only significant for hypopigmented skin lesions consistent with her medical history of vitiligo and a soft pan systolic grade II murmur. She had normal blood pressure readings for age, height, and sex.

Initially, the child underwent a workup to rule out bacterial infectious causes, glomerular, and autoimmune-related glomerulonephritis. Automated urinalysis and microscopy using Iris iQ200 did not detect the schistosome eggs. A history of travel to a schistosomiasis endemic area was elicited on a subsequent visit, prompting a thorough ova and parasite examination of the urine. Manual urine microscopy on a centrifuged urine sample confirmed the diagnosis of *S. hematobium* infection.

## Investigations

Blood and urine tests are presented in Table 1. Kidney and bladder ultrasound was significant for bladder debris, otherwise unremarkable. Kidney biopsy pathology result was reported as normal light (H&E, special stains) and negative immunofluorescence (EM was deferred due to high cost to the patient).

**Table 1:** Blood and urine laboratory test results.

Blood			Urine		
Test	Result	Reference	Test	Result	Reference
Hemoglobin	122 g/L	102–127		**Urine dipstick and automated microscopy**	
WBC	7.8 × 10^9^/L	4.9–13.2	Color	Brown	
Platelet	298 × 10^9^/L	189–394	SG	1.025	1.005–1.035
Na	138 mmol/L	135–145	Bilirubin	1+	Negative
K	4.1 mmol/L	3.5–5.2	pH	8.5	5.0–8.0
Cl	105 mmol/L	98–116	Blood	3+	Negative
Bicarbonate	23 mmol/L	21–31	Glucose	Negative	Negative
BUN	4.0 mmol/L	3.5–8.3	Ketones	1+	Negative
Cr	25 μmol/L (L)	26–51	Protein	3+	Negative
Bilirubin	10 μcmol/L (H)	2–7	WBC	100/HPF (H)	0–5
Alk Phos	214 IU/L	134–315	RBC	>100/HPF (H)	0–5
Total protein	69 g/L	62–75			
Albumin	42 g/L	35–46			
ALT	20 IU/L	12–27			
AST	34 IU/L	24–46		**Urine chemistry**	
Ca	2.41 mmol/L	2.32–2.64	Urine creatinine	11.0 mmol/L	
C3	137 mg/dL	86–166	Urine protein	2,469.0 mg/L	
C4	34.8 mg/dL	13–32	Urine protein/creatinine	224 mg/mmol Cr (H)	0–20
ANA	Negative			**Urine culture**	
ANCA	Negative		Urine culture	No growth	
Anti-GBM	Negative			**Urine ova and parasite**	
Rheumatoid factor	Negative		Urine ova and parasite	Ova of *Schistosoma hematobium* seen	Negative

**Figure 2:**
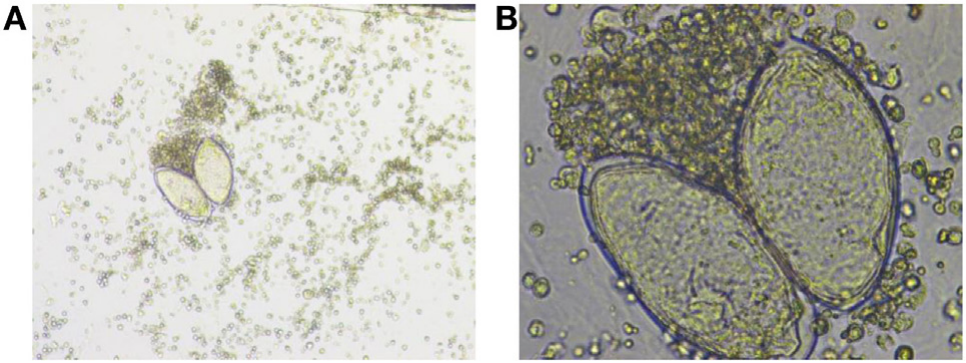
**(A)** Low-power and **(B)** high-power photos showing *Schistosoma hematobium* eggs in a urine sample from our patient. Courtesy of Nazik Elamin, MIBMS from Sidra Medical and Research Center microbiology lab.

Microscopic identification of the parasite eggs in urine by urine microscopy is the most practical method for schistosomiasis diagnosis. It is relatively simple, inexpensive, and considered to be the gold standard for diagnosis (3), Figures 2A,B.

Schistosoma eggs are passed intermittently in small amounts and may not be detected, so it may be necessary to perform a serological test to confirm the diagnosis. However tissue biopsy (rectal biopsy for all species and biopsy of the bladder for *S. hematobium*) may demonstrate eggs when stool or urine examinations are negative (4).

Serological testing for antischistosomal antibody is indicated in people with suspected schistosome infection who have traveled to an endemic area, but their urine and stool is negative for the parasite eggs. The serological testing is also indicated for diagnosis of travelers or immigrants from endemic areas who have not been treated appropriately for schistosomiasis in the past (4).

## Differential Diagnosis

A long list of etiologies falls under the differential diagnosis of gross hematuria in children. To help localize the origin of hematuria, the healthcare provider should start by obtaining a detailed history to differentiate red, fresh blood in the urine from cola-colored urine, and whether the hematuria is at the beginning, throughout, or is terminal during micturition, persistent or intermittent, and if the hematuria is associated with flank pain? A medication and food history can help to rule out dye or pigmenturia. An infectious etiology is usually accompanied by fever, dysuria, and is shorter in duration. A glomerular origin of the gross hematuria is usually accompanied by signs and symptoms of fluid overload, high blood pressure, and proteinuria and maybe preceded by history of upper respiratory tract infection or a skin infection.

The medical history should also include asking the patient about any history of trauma, personal and family history of blood disorders (bleeding tendency and sickle cell disease), malignancies, and history of travel.

The physical examination should include the inspection of the external genitalia for any signs of trauma as well as to look for any signs of systemic illnesses that may present with glomerulonephritis such as joint swelling, redness, facial rash, petechial rash, and generalized swelling.

The laboratory and imaging workup should be guided by the history and physical examination.

## Discussion

Our patient received three doses of praziquantel at 20 mg/kg given in 24 h as the treatment of choice for *S. hematobium*. In patients with pretreatment positive schistosome eggs, as in our patient, a repeat urine microscopic examination is recommended in 1–2 months to confirm successful cure (4). A repeat urinalysis and microscopic examination were performed in our patient and results came back negative for ova and parasite.

There are an estimated 240 million people infected with one of the major schistosomes, more than 700 million people live in endemic areas, and 200 thousand annual deaths are attributed to schistosomiasis in sub-Saharan Africa (5).

**Figure 3:**
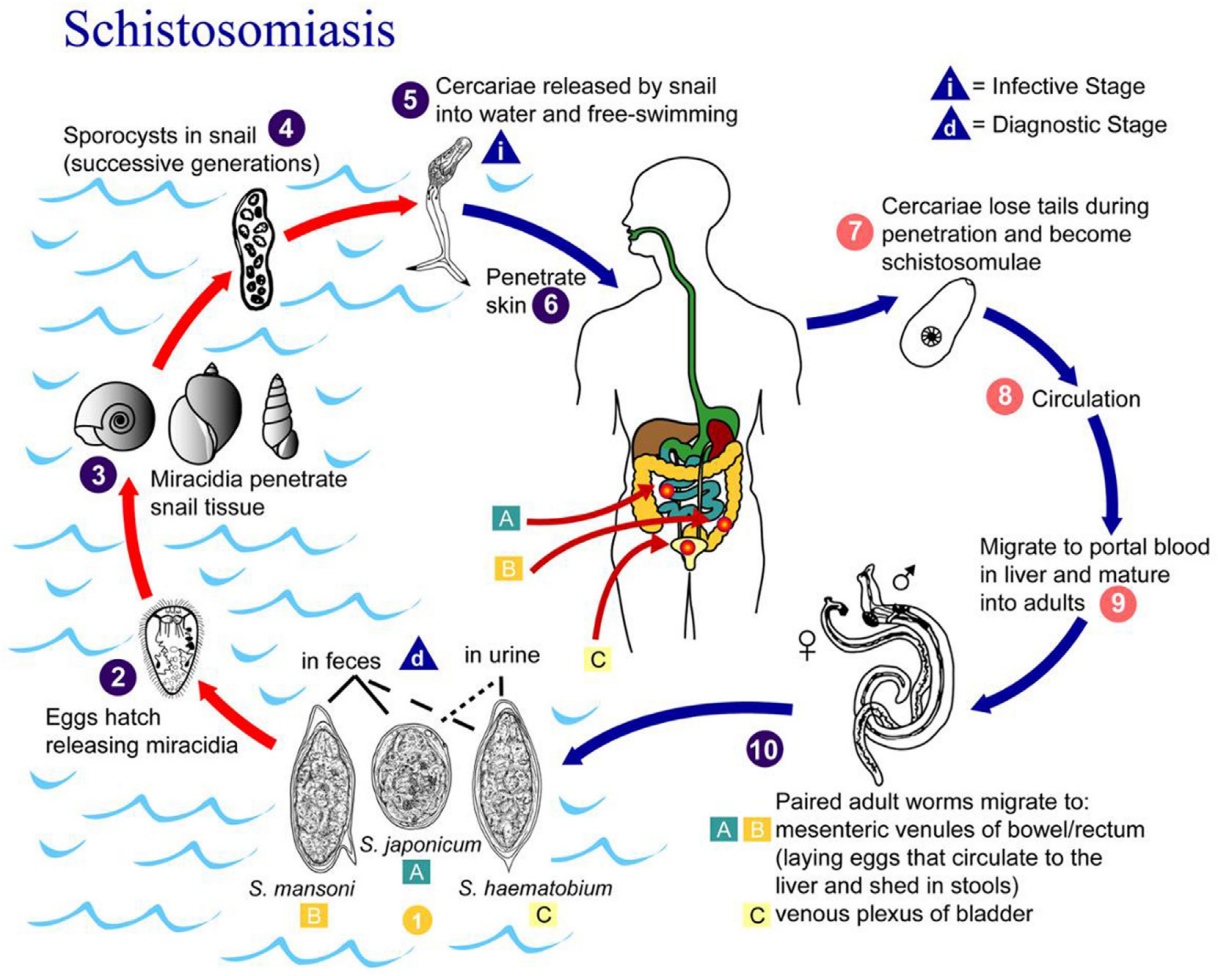
Life cycle of *Schistosoma hematobium, Schistosoma mansoni*, and *Schistosoma japonicum*. Source: CDC, Atlanta, GA, USA (7).

Schistosomiasis progresses in three distinct phases: acute, chronic, and advanced disease (6). A maculopapular rash may arise at the site where the schistosome cercariae penetrate the skin. In the acute phase, symptoms of schistosomiasis are caused by the body’s reaction to the worms’ eggs, which are deposited by adult worms in the blood vessels surrounding the bladder or the intestine, Figure 3 (7). Symptoms may include fatigue, malaise, fever, cough, diarrhea (with or without the presence of blood), hematuria (*S. hematobium*), and abdominal pain. The chronic and advanced disease phases are associated with a chronic local inflammatory response to schistosome eggs, which may lead to inflammatory and obstructive disease in the urinary system (*S. hematobium*).

In urogenital schistosomiasis, the patient may present with urogenital bleeding, genital lesions, and nodules. Long-term consequences of chronic infections include infertility, bladder and urethral fibrosis, obstructive uropathy, and hydronephrosis; moreover, bladder cancer has been reported as a possible late-stage complication (8). Renal involvement in patients with schistosomiasis is the result of both direct invasion of the kidney or the urinary tract and/or immune-mediated renal injury secondary to host–parasite interaction with renal involvement. Renal findings can range from abnormal urinary sediment to significant proteinuria, acute kidney injury, and nephrotic/nephritic syndromes (mesangiocapillary glomerulonephritis, membranous glomerulonephritis, and focal segmental glomerulosclerosis) in some cases (9).

Our patients’ symptoms were likely secondary to her urinary bladder infection. A urine protein to creatinine ratio of 224 was attributed to the grossly bloody urine as well as the inflammation in her bladder. Her kidney biopsy was reviewed again after the schistosomiasis diagnosis; no glomerular or interstitial pathological findings of schistosomiasis infection were identified. Our patient maintained a normal serum albumin level throughout her illness.

After our initial extensive workup, a history of travel to Malawi prior to the appearance of our patient’s symptoms leads us to rethink the differential diagnosis of gross hematuria. Clinicians need to be aware of the automated urine analysis and microscopy limitations and to perform a manual urinalysis on a freshly voided and centrifuged urine sample in patients with gross hematuria. Careful review of the travel and residence history is critical, not only to determine whether schistosome infection is likely, but also to determine species that may be causing the infection.

## Ethics Statement

Written informed consent was obtained from the patients’ family prior to presenting the case.

## Author Contributions

IS is the sole author of this case report.

## Conflict of Interest Statement

The author declares that the research was conducted in the absence of any commercial or financial relationships that could be construed as a potential conflict of interest. The authors confirm no ethical conflict with the patient.

## References

[1] IARC Working Group on the Evaluation of Carcinogenic Risks to Humans. Biological agents. Volume 100 B. A review of human carcinogens. IARC Monogr Eval Carcinog Risks Hum (2012) 100(Pt B):1–441. PMC4781184 23189750

[2] Gryseels B Polman K Clerinx J Kestens L. Human schistosomiasis. Lancet (2006) 368(9541):1106–18. 10.1016/S0140-6736(06)69440-3 16997665

[3] Bamgbola OF. Urinary schistosomiasis. Pediatr Nephrol (2014) 29(11):2113–20. 10.1007/s00467-013-2723-1 24469437

[4] CDC. Available from: https://www.cdc.gov/parasites/schistosomiasis/health_professionals/index.html#dx

[5] WHO. Schistosomiasis. Available from: http://www.who.int/mediacentre/factsheets/fs115/en/

[6] Gray DJ Ross AG Li YS McManus DP Diagnosis and management of schistosomiasis. BMJ (2011) 342:d2651 10.1136/bmj.d2651 21586478 PMC3230106

[7] CDC. Available from: https://www.cdc.gov/dpdx/schistosomiasis/index.html

[8] Felix AS Soliman AS Khaled H Zaghloul MS Banerjee M El-Baradie M The changing patterns of bladder cancer in Egypt over the past 26 years. Cancer Causes Control (2008) 19(4):421–9. 10.1007/s10552-007-9104-7 18188671 PMC4274945

[9] Kamath N Iyengar A. Infections and the kidney: a tale from the tropics. Pediatr Nephrol (2017) 1–10. 10.1007/s00467-017-3785-2 28879600

